# The Lymphatic Headmaster of the Mast Cell-Related Splanchnic Inflammation in Portal Hypertension

**DOI:** 10.3390/cells8070658

**Published:** 2019-06-29

**Authors:** Maria-Angeles Aller, Javier Blanco-Rivero, Natalia Arias, Luis Santamaria, Jaime Arias

**Affiliations:** 1 Department of Surgery, School of Medicine, Complutense University of Madrid, 28040 Madrid, Spain; 2 Department of Physiology, School of Medicine, Autonomous University of Madrid, 28049 Madrid, Spain; 3 Instituto de Investigación Biomédica La Paz (IdIPAZ), 28046 Madrid, Spain; 4 Centro de Investigación Biomédica en Red (Ciber) de Enfermedades Cardiovasculares, 28029 Madrid, Spain; 5 Department of Basic and Clinical Neuroscience, Institute of Psychiatry, Psychology and Neuroscience, King’s College London, London WC2R 2LS, UK; 6 INEUROPA (Instituto de Neurociencias del Principado de Asturias), 33003 Oviedo, Spain; 7 Department of Anatomy, Histology and Neuroscience, School of Medicine, Autonoma University of Madrid, 28029 Madrid, Spain

**Keywords:** lymphatics, mast cells, peroxisomes, portal hypertension

## Abstract

Portal hypertension is a common complication of liver disease, either acute or chronic. Consequently, in chronic liver disease, such as the hypertensive mesenteric venous pathology, the coexisting inflammatory response is classically characterized by the splanchnic blood circulation. However, a vascular lymphatic pathology is produced simultaneously with the splanchnic arterio-venous impairments. The pathological increase of the mesenteric venous pressure, by mechanotransduction of the venous endothelium hyperpressure, causes an inflammatory response involving the subendothelial mast cells and the lymphatic endothelium of the intestinal villi lacteal. In portal hypertension, the intestinal lymphatic inflammatory response through the development of mesenteric-systemic lymphatic collateral vessels favors the systemic diffusion of substances with a molecular pattern associated with damage and pathogens of intestinal origin. When the chronic hepatic insufficiency worsens the portal hypertensive inflammatory response, the splanchnic lymphatic system transports the hyperplasied intestinal mast cells to the mesenteric lymphatic complex. Then, an acquired immune response regulating a new hepato-intestinal metabolic scenario is activated. Therefore, reduction of the hepatic metabolism would reduce its key centralized functions, such as the metabolic, detoxifying and antioxidant functions which would try to be substituted by their peroxisome activity, among other functions of the mast cells.

## 1. Portal Hypertension and Splanchnic Lymphatic Pathology

Portal hypertension is defined as the pathological increase of portal pressure, which is determined by a hepatic venous pressure gradient (HVPG) greater than 5 mmHg, with complications arising once this pressure exceeds 10 mmHg. As a result of elevated pressures within the portal vein, several complications can arise, including the development of esophageal and gastric varices, ascites, hepatic encephalopathy, as well as complications secondary to circulatory dysfunction, including hepatorenal syndrome, portopulmonary syndrome, and hepatopulmonary syndrome. In turn, the etiology of increased portal resistance is commonly categorized according to the anatomical location in terms of pre-hepatic, intra-hepatic, and post-hepatic causes [1]. Intrahepatic portal hypertension is the most frequent in the clinical area and is principally produced by toxic substances (alcoholic liver disease), chronic infections due to HBV and/or HCV, and metabolic pathologies (Non-alcoholic liver steatosis, NASH) [2,3].

Pathological portal pressure increases when it is related to liver disease, producing systemic and splanchnic impairments that together could be considered a syndrome; that is, the portal hypertensive syndrome.

Until now, the study of portal hypertension has mainly focused on the blood vascular ethiopathogeny in detriment of the lymphatic vascularization. Consequently, hyperdynamic circulation and excessive angiogenesis are key blood vascular characteristics of portal hypertension in the splanchnic area. Excessive angiogenesis is localized in the macrocirculation, with the development of porto-systemic collateral circulation, with an increase of the mucosa and submucosa blood vascularization in the gastrointestinal layer. Hence, the enteropathy developed in portal hypertension has been named portal hypertensive vascular enteropathy [4].

Furthermore, the leading role of the splanchnic venous vascularization in portal hypertension is easily demonstrable in the experimental models. In particular, in the model of prehepatic portal hypertension in the rat by partial ligation of the portal vein, it is possible to observe in the short-term the great splanchnic angiogenic response that is produced. Thus, through a laparotomy performed in the early evolutive stages, the existence of porto-systemic collateral circulation, of paraoesophageal, splenorenal and pararectal types, can be observed. This portal hypertensive enteropathy is characterized by a great dilation and tortuosity of the mesenteric vein branches. Taken all together, these alterations represent the ability of the splanchnic venous pressure to stimulate the blood vascular endothelium proliferation [5]. Since the increase of blood pressure in portal hypertension is attributed to the mechanical energy increase, it is obvious that this type of energy through the mechanism known as the mechanotransductor, has the ability to induce the abovementioned angiogenic stimulus early. In turn, it has been shown that excessive endothelial mechanotransduction is a proinflammatory stimulus. Therefore, it is proposed that the endothelial pathology of portal hypertension is inflammatory [6].

Particularly, the splanchnic post-capillary venule endothelium has great sensibility to the portal pressure increase, when the hypertension is secondary to a liver disease. This fact allows for an early change in the endothelium phenotype, which expresses an inflammatory phenotype before developing hepatic insufficiency [7].

However, portal hypertension also induces the splanchnic lymphatic pathology, although this alteration has been undervalued until recently because these macroscopic alterations are not as noticeable as the blood ones. A more detailed study of the splanchnic lymphatic macro- and microcirculation would allow us to confirm the key role of this lymphatic vascular pathology in terms of the blood vascular pathology in portal hypertension.

## 2. The Mast Cell as Mediator of the Splanchnic Lymphatic Pathology in Portal Hypertension

Since the mast cells are located in close proximity to the blood and lymphatic microcirculation, through a paracrine mechanism, they can influence the endothelial cells of both the blood and lymphatic circulatory systems. In addition, mast cells can act on other cell residents in this environment, including pericytes, fibroblasts, and other immune cells, both resident and recruited. [8]. Particularly, the inflammatory alterations induced by mechanical energy in portal hypertension could be influenced by endothelial mechanotransduction in the expression of a particular inflammatory phenotype in the resident mast cells. The functional heterogeneity and plasticity of the mast cells would condition the heterogeneity of the blood and lymphatic endothelial cells that it influences [9]. This would explain why the functional endothelial cell-mast cell coupling plays the key role in the splanchnic inflammatory impairments induced by mechanotransduction in portal hypertension. In experimental and clinical portal hypertension, an increase of the splanchnic mast cells as well as the changes of their phenotype have been demonstrated [10,11,12]. The increase of the mast cells number in the hepato-intestinal axis when a pathological increase of portal hypertension is produced, suggests that portal hypertension is the cause of mastocytosis. In turn, the change of phenotype of these cells, considered immune cells in portal hypertension, was originally demonstrated in rodents [13]. Two types of mast cells, the connective tissue mast cells (CTMC), and the mucosal mast cells (MMC) are well defined in these mammals [14]. These two populations of mast cells differ in many aspects, including their responses to drugs and secretagogues [15]. Thus, CTMC are sensitive to depletion by compound 48/80,31 whereas dexamethasone prevents MMC activation. Cytoplasmic granules of CTMC are rich in heparin and histamine, whereas in MMC the cytoplasmic granules contain the proteoglycan chondroitin-6-suphate, but low concentrations of histamine [16].

Another difference between MMC and CTMC is the pattern of Arachidonic acid-derived metabolites. The predominant Arachidonic acid metabolite of CTMC is Prostaglandin D2, whereas MMC generate predominantly lipoxygenase products such as Leukotriene D4 and Leukotriene E4 [14]. In addition, the serinproteases of the mast cells have been classified in chymases and tryptases. All the proteases in the rat are chymases, in MMC rat mast cell protease 2 (RMCP-2), mucosal mast cell protease 5 (MMCP-5), and Carboxipeptidase, while CTMC produces RMCP-1 [17]. This “plasticity” of multiple aspects of the mast cell’s results in the development of phenotypically distinct populations of mast cells in different anatomical sites, as well as in different animal species, is called “mast cell heterogeneity” [18].

Depending on the anatomical site in which the mast cells reside and/or the biological processes in which they participate allows for greater flexibility and diversity of mast cell responsiveness [19].

**Figure 1:**
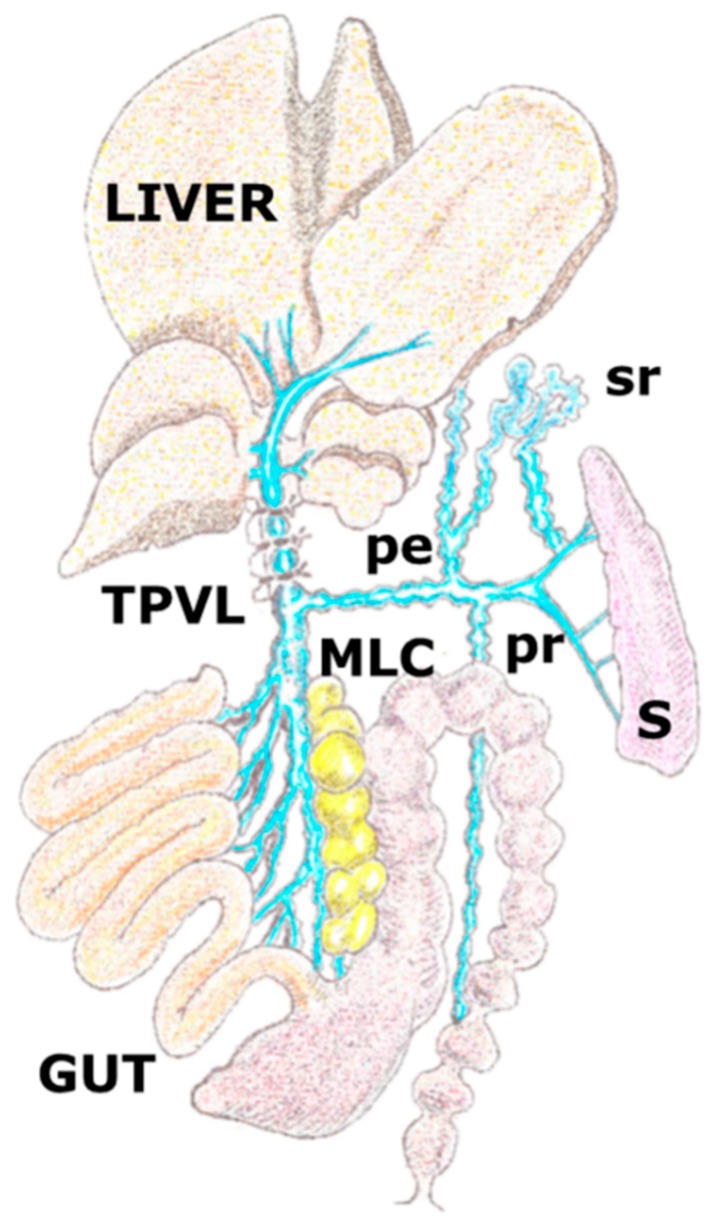
Graphic representation of the porto-systemic collateral circulation in the rat after performing a partial portal vein ligation. The triple partial portal vein (TPVL) ligation technique has been used to increase resistance of venous portal flow and subsequently, the portal hypertension degree and therefore the related splanchnic inflammatory response. Collateral circulation with paraoesophageal (pe), splenorenal (sr) and pararectal (p) vessels. MLC: mesenteric lymphatic complex; S: spleen.

The stenosing ligation of the portal vein, also named partial portal vein ligation (Figure 1) [20], is an experimental model in rodents used to study portal hypertension without associated liver disease. The portal hypertension obtained this way progresses with a significant increase of mast cells either in the liver [21] or in the hepato-intestinal axis [10,11,13,22]. That’s why it has been suggested that these cells would be involved in the exacerbated inflammatory angiogenic response developed in the abovementioned axis. A great number of mediators from the mast cells have angiogenic functions, especially histamine, vascular endothelial growth factor (VEGF), and nitric oxide (NO). However, the mast cells also have the ability to produce lymphangiogenic factors, principally VEGF-A, VEGF-B, VEGF-C, and VEGF-D [23,24].

Therefore, it should be noted that lymphangiogenesis must be considered among the pathological consequences of the portal hypertensive splanchnic mastocytosis. In addition, the mast have the abilities to produce vasodilation and increased permeability of the vascular, blood and lymphatic, endothelium. These actions are mediated by the production and release by the mast cells of VEGFs and tryptase, as an activator of protease activated receptors (PAR) and Peroxisome proliferator-activated receptor-gamma (PPAR-γ), [9,25].

**Figure 2:**
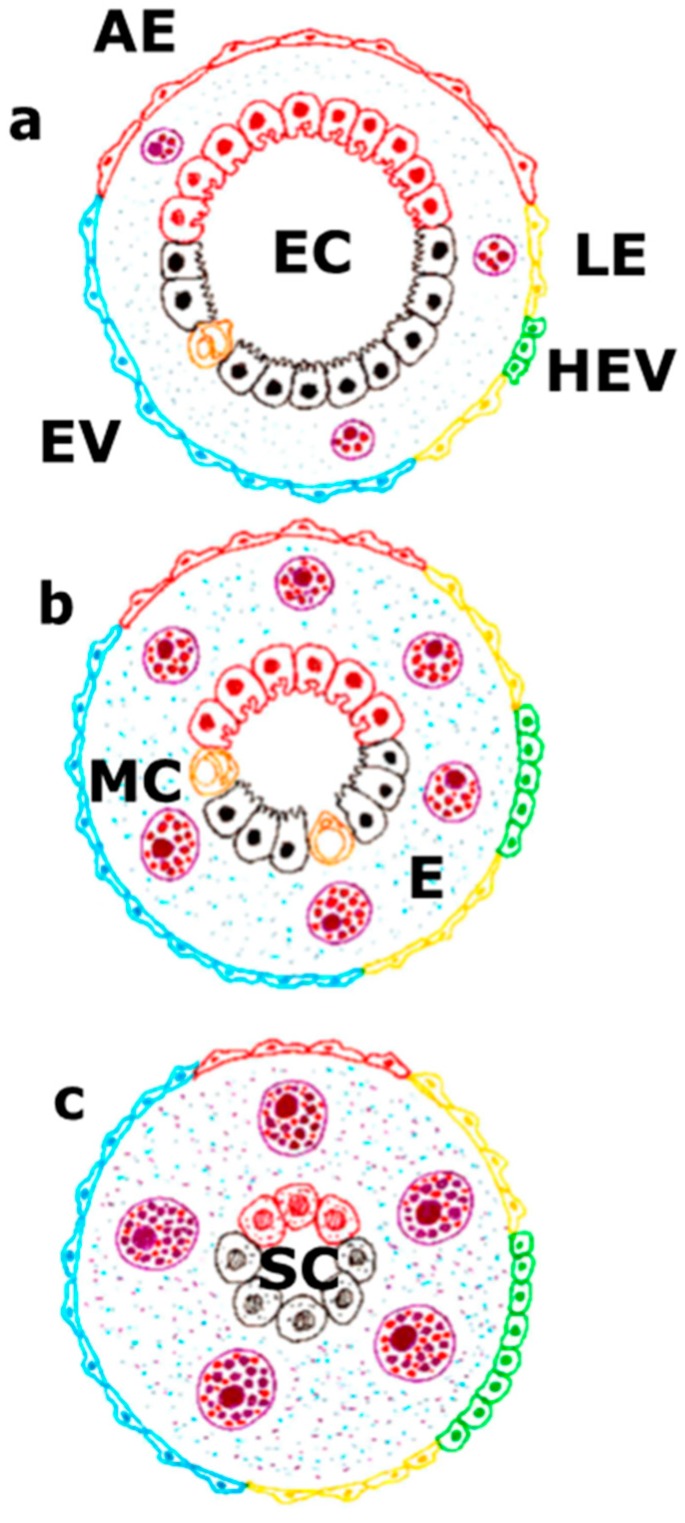
The vascular endothelium takes part either in the physiological situation (**a**), in the splanchnic inflammatory response related to portal hypertension (**b**) or in the portal hypertension associated with chronic liver disease (**c**). In the physiological situation, a balance between the extent of the arterial endothelium, venous endothelium, including high endothelial venules, and lymphatic endothelium exists. This fact is associated with the presence of a specialized hepato-intestinal epithelium. In this case, the mast cells would make a basic function of maintaining this endothelium-epithelium coupling. On the contrary, during the progressive development of portal hypertension, the splanchnic mast cell number increases. Due to their inflammatory activation, they cause progressive interstitial edema. This impairment coexists with an increase of the leading role of the splanchnic endothelial lymph-venous at the expense of the arterial endothelium, as well as with a reduction of the specialized functions of the hepato-intestinal epithelium. Lastly, when portal hypertension is associated with a chronic liver disease, the great predominance of the lymph-venous endothelium would correlate with a functional activation of the mast cells that try to compensate the functional hepatic deficit and preserve the hepato-intestinal survival mechanisms, i.e., the stem cells. In turn, the splanchnic interstitial edema would reach its maximal expression. AE: arterial endothelium; E: edema; EC: hepatointestinal epithelial cells; HEV: high endothelial venule; LE: lymphatic endothelium; MC: mast cell; SC: stem cells; VE: Venule endothelium.

The blood vasodilation, by increasing the blood flow speed, reduces the oxygen uptake by the intestinal layer cells, while the increase of the blood endothelium permeability causes edema (Figure 2). So, the intestinal epithelial cells, which normally obtain energy through oxidative phosphorylation, would be deprived of obtaining oxygen by a dual mechanism. Firstly, this would occur because the hyperdynamic circulation prevents its blood uptake, and secondly, due to the increased distance to the blood capillaries cause d by the edematous interstitium.

**Figure 3:**
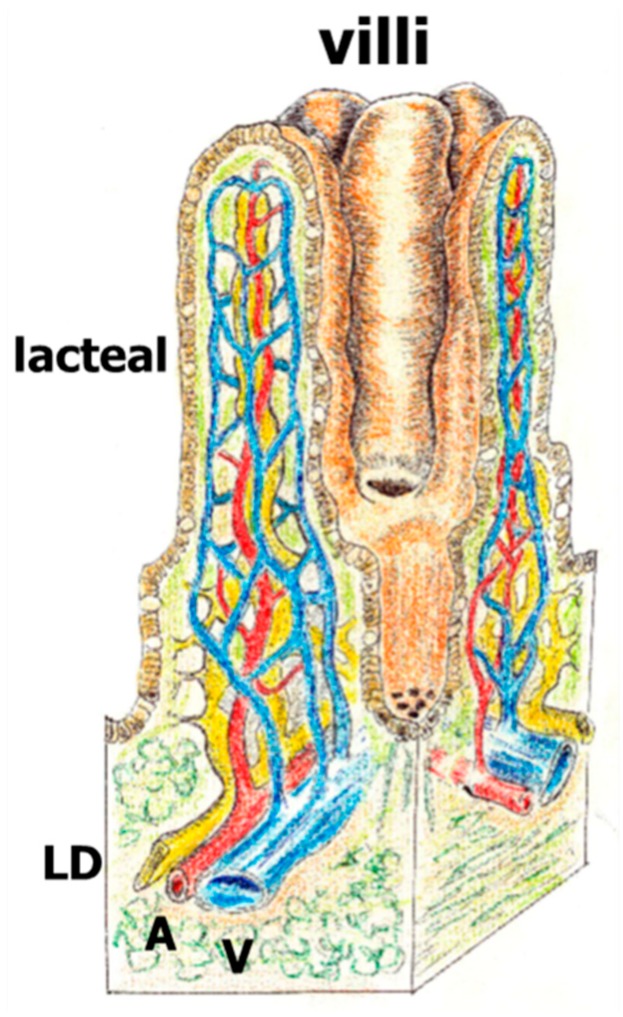
Draft of the intestinal villi with lymphatic lacteal and lymphatic ducts (LC: yellow), artery (A: red) and vein (V: blue).

The splanchnic lymphatic system that is understood more today is the intestinal lymphatic system since it carries the products derived from food digestion. In fact, its principal function is the absorption and transport of dietary fat. Spontaneous lacteal contraction, in concert with adjacent smooth muscles, is essential for drainage of dietary lipids. Thus, the lymphatic pumping activity is regulated by intraluminal pressure and wall shear stress via differential release of prostaglandins, NO and histamine [26]. Lymphatic endothelial cell differentiation in vertebrates occurs primarily from venous endothelial cells, although a mesenchymal origin has also been shown [27]. Lymph from the gastro-intestinal and lumbar region drains into the cisterna chyli at the posterior end of the thoracic duct and reaches the circulation in the subclavian vein. In the intestine, lymphatic capillaries, or lacteals, are located exclusively in intestinal villi, whereas collecting lymphatic vessels are present in the mesentery (Figure 3) [26].

In portal hypertension the lymphatic alteration related to the blood vascular impairments is the basis of the creation of an endothelial blood-lymphatic pathological coupling mediated by the splanchnic mast cells. In this way, the hypoxic conditions imposed by the blood vascular circulation in the gastrointestinal tract could be responsible for a pathological lymphatic circulation development. So, lymphatic capillaries facilitate the interstitial drainage and transport either resident immune cells or recruited ones. The mediators released by the mast cells, especially heparin and VEGFs, produce an increase of the lymphatic endothelial permeability as well as lymphangiogenesis [28,29]. It has also been described that a deficit of the lymphatic vessel contractility is associated with the increase of lymphatic flow, which strengthens the interstitial edema [26,29,30].

The microcirculatory impairments in portal hypertension are secondary to the endothelial metabolic alterations. Although the blood endothelial metabolism has been thoroughly studied in the angiogenic inflammatory response [27], the lymphatic endothelial metabolism during the inflammatory response is still the great unknown today. However, it could be supposed that the metabolic alterations of the lymphatic endothelium in inflammation could be similar to the blood endothelial metabolism, although with the specificity of the lymphatic endothelium. If so, the metabolic mediation of the mast cells would integrate the functions of the blood-lymphatic endothelial coupling. The mast cells could participate by supporting basic substrates for the endothelial metabolism [31]. In particular, through the support of their precursor amino acids, namely histidine and tryptophan, the corresponding biogenic amines, histamine, and serotonin respectively, could be synthesized by the mast cells [32]. Histidine is an amino acid that plays a key role in providing methyl donors for DNA and protein methylation and, therefore, it is a potential producer of epigenetic changes modifying the activation of certain genes in the endothelium [33,34]. In turn, alanine is a non-essential amino acid that is involved in the metabolism of tryptophan, which can also be a methyl donor [34,35]. Of note, to the potential ability of these amino acids to make epigenetic changes, their demonstrated protecting function as part of the solution for hypothermic preservation of organs in transplantation could be added [36].

Therefore, one of the basic mechanisms that induces gastrointestinal lymphangiogenesis either in portal hypertension or in liver cirrhosis could not be excluded as epigenetic, even mediated by mast cells [29]. Hence, the mast cells’ potential use of the biogenic amine precursor amino acids in the splanchnic inflammation related to portal hypertension suggests that the abovementioned amino acids serve as a parametabolic link between metabolism and epigenetics [33]. In addition, these amino acids could be used in the preservation of the splanchnic tissues and organs that are edematous and hypoxic and, consequently, have an anaerobic metabolism.

## 3. The Mast Cell and the Lymphatic Gut-Liver Axis

The splanchnic lymphatic pathology related to portal hypertension could be relative to the inflammatory response mediated by mast cells. In this way, the mast cells are a sensor of the portal venous pressure increase.

### 3.1. Mechanotransduction, Liver Disease and Mast Cells

In essence, the mast cells are a physic-chemical sensor because of the mechanotransductor stimulus received, and therefore a metabolic stimulus is added due to the liver insufficiency. It’s possible that in the presence of these etiological factors, the mast cells, thanks to their great plasticity [19,37], simultaneously express different phenotypes. Thus, these two different expressed phenotypes are a portal hypertensive phenotype and a compensating phenotype of the hepatic functional deficit. In the case in which portal hypertension may have intrahepatic origin, which is common in chronic hepatic insufficiency, a severe and progressive metabolic insufficiency is associated. This metabolic hepatic insufficiency is evaluated through the Child and the Model For End-Stage Liver Disease MELD scores [38,39]. The Child–Pugh (CP) classification is one of the most common bedside tools utilized in medicine for gauging the severity of liver disease and estimating prognosis in the patient with cirrhosis. CP is a composite ordinal score consisting of three laboratory-based biomarkers (serum albumin levels, serum bilirubin levels, and prothrombin time) and two clinically assessed variables (presence/degree of ascites and presence/degree of hepatic encephalopathy) [38]. The present version of MELD score incorporated only 4 objective variables, including total bilirubin, creatinine, international normalized ratio (INR) and serum sodium level [39]. Therefore, this noxious pathological liver-related factor would condition the evolution of the lymphatic impairment of portal hypertension.

### 3.2. Splanchnic Mast Cells and Lymphangiogenesis

**Figure 4:**
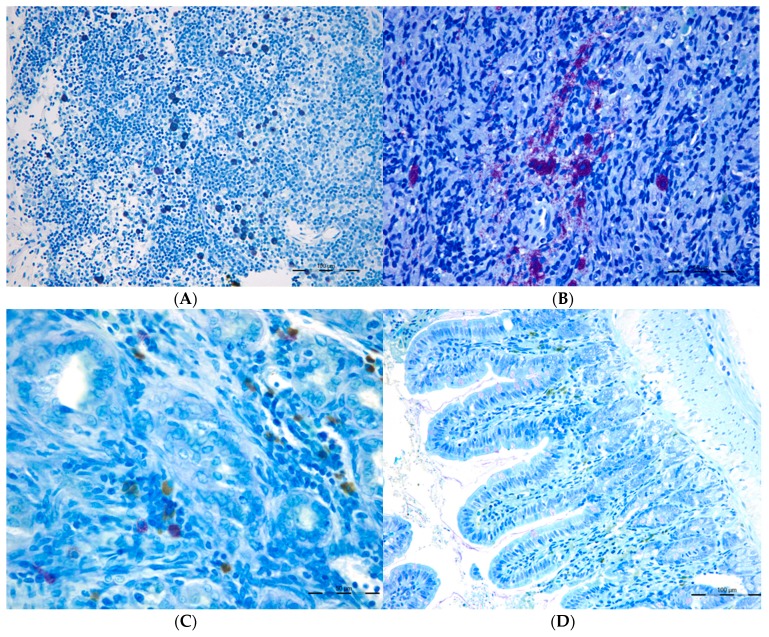
Connective Tissue mast cells (CTMC) were identified after stained with a 0.5% toluidine blue solution transversal sections of (**A**) the lymphatic nodes and in (**B**) spleen as granulated violet/blue cells) in cholestatic rats. Mucosal mast cells (MMC), rat mast cell protease 2 (RMCP-II), green immunopositive cells in (**C**) the liver and (**D**) in the ileum of microsurgical cholestatic rats. Immunodetection of RMCP-II, specific marker of mucosal mast cells (MMC) in the rats, was carried out using a specific rat monoclonal antibody (1:500; Moredun Animal Health, Edinburgh, UK). The size of the calibration bars throughout all images was 100 µm.

**Figure 5:**
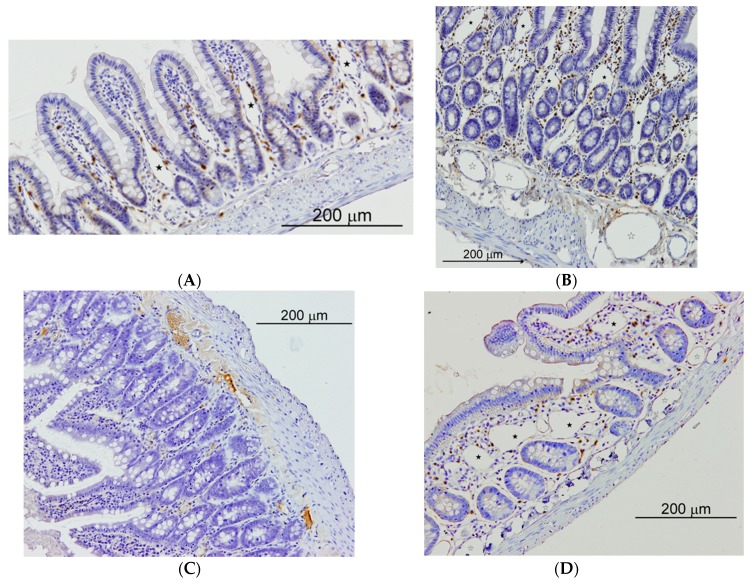
Small intestine of sham operated (SO) and microsurgical cholestatic-rats (MC) immunostained with rabbit polyclonal anti-LYVE-1, dilution 1:500 (Abcam; Cambridge, UK), a specific marker of lymphatic endothelium. (**A**) image of a duodenum SO-rat: some small lymphatics lumen are observed in the villi axis that are scarcely enlarged (full star); (**B**) duodenum from a rat with MC: abundant and enlarged lymphatics are observed either in the mucosa (full stars) as in the submucosa (empty stars); (**C**) Image of an ileum SO-rat: any lymphatic lumen is observed in the villi; (**D**) ileal image from an animal with MC showing a relevant enlargement of lymphatic microvessels either in the mucosa (full stars) as in the submucosa (empty stars). The size of the calibration bars throughout all images was 200 µm.

We’ve demonstrated the existence of a splanchnic increase in the number, as well as a phenotype change of the mast cells (CTMC and MMC) in portal hypertension associated with the cholestatic hepatic insufficiency that also shows an excessive intestinal lymphangiogenic response (Figure 4 and Figure 5) [40]. In these cholestatic rats, CTMC and MMC showed an increase in the mesenteric lymphatic complex. MMC increased in the liver and ileum, and both MMC and CTMC increased in the spleen [40]. In a stereological study, by immunolabelling the intestinal lymphatic vessels with LYVE-1, a specific marker of lymphatic endothelium, we’ve shown that the lymphatic portal hypertensive enteropathy is characterized by lymphatic length density and microvessel volume fraction increase. These lymphatic impairments are more marked in the duodenum and ileum. This study demonstrates the existence of an increased lymphangiogenesis in both sides of the small bowel associated with liver fibrosis (Figure 5). It also suggests the development of a splanchno-systemic lymphatic collateral circulation in portal hypertension.

So, intestinal lymphangiogenesis is more severe in the duodenum and ileum in cholestatic rats, and this finding suggests the development of a lymphatic collateral drainage from both ends of the gastrointestinal tract. This is the reason why the development of a mesenteric-systemic lymphatic circulation would be added to the existence of a porto-systemic blood circulation. The systemic spreading of intestinal inflammatory mediators through these mesenteric-systemic lymphatic vessels would have a similar pathogenic result to what their already thoroughly studied spreading mediators have through the porto-systemic blood bypass. If so, not only microorganisms and their metabolites (PAMPs) of intestinal origin, but also other intestinal proinflammatory mediators, like DAMPs, cytokines and chemokines, would reach, through the mesenteric-systemic lymphatic bypass, tissues and organs far off the gastrointestinal tract. This systemic flooding of proinflammatory mediators secondary to the splanchnic lymphatic disfunction would be worsened by the coexistence of the immunosuppression in patients with liver diseases.

### 3.3. Mast Cells Compensating Chronic Liver Disease

**Figure 6:**
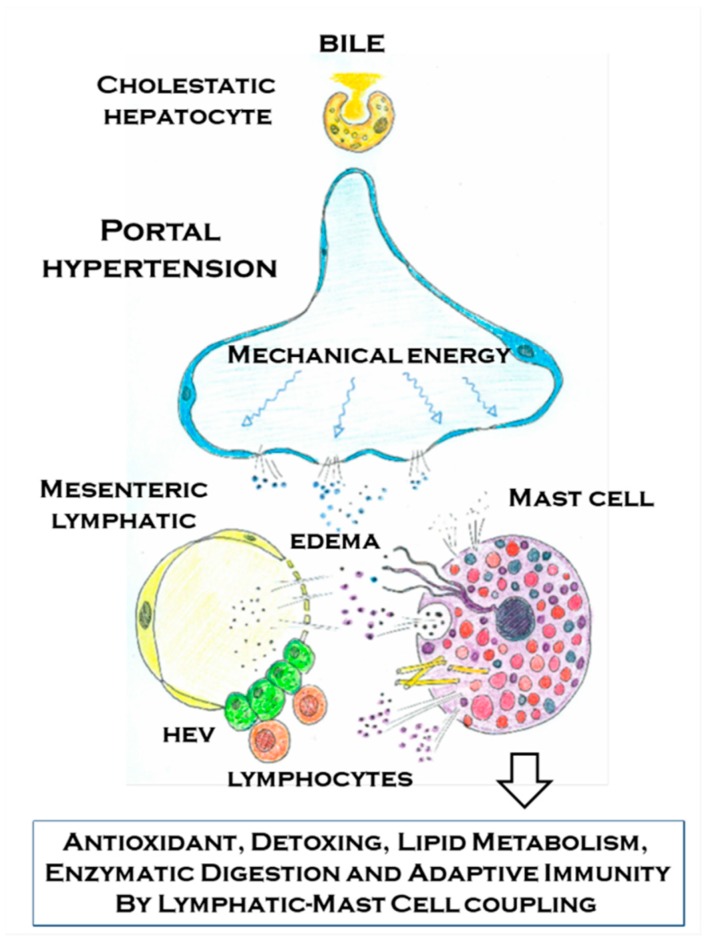
Schematic representation of the relationship that is established in portal hypertension associated with cholestatic chronic liver diseases between the mesenteric venous portal and lymphatic endothelium with the mast cell. The sensor ability of the mast cells to chemical (damage associated molecular pattern and pathogen associated molecular pattern) and physical (mechanical energy) stimuli allows it to selectively react by means of its multiple mechanisms to the abovementioned stimulus in portal hypertension. The mast cells mechanisms of action include degranulation, exosomes/extracellular vesicles, tunneling nanotubes, extracellular trap formation and mast cells migration to the mesenteric lymph nodes, where they reach the high endothelial venules (HEVs), and then they would activate the adaptive immunity. The mast cells through these mechanisms would try to antagonize some of the impairments that are typical of portal hypertension associated with chronic liver disease. Between the protecting functions of the mast cells and their ability to antagonize the oxidative stress, the toxic intestinal substances and the immunologic injury stand out.

The splanchnic mast cells, behaving as a metabolic sensor in chronic hepatic insufficiency, would have a modulating behavior of these metabolic alterations [41]. In particular, two functions that are severely impaired in chronic liver disease, like the detoxing function made by the Kupffer cells, and the antioxidant function, mediated by the peroxisomes [42], could be partially improved by the compensating actions of the mast cells [43] (Figure 6). Furthermore, the splanchnic mast cells could develop metabolic functions that adapt to this new metabolic environment. Therefore, the ethopathogenic factor most relevant to liver disease that worsens the splanchnic lymphangiogenic disfunction secondary to portal hypertension would be the hepatic insufficiency [44].

The liver diseases, especially if they are chronic with cirrhosis, are characterized by chronic necroinflammatory and fibrogenetic processes. The key impairments shown in cirrhosis are the conversion of normal liver architecture into structurally abnormal nodules, dense fibrotic septa, concomitant parenchymal exhaustion, and collapse of the liver tissue [45]. The unstructured hepatic parenchyma by fibrosis would obstruct the liver lymphatic drainage; that is, the 25–50% of the lymph that reaches the thoracic duct. Consequently, this hepatic lymphatic stasis has been involved in the ascites production [46]. In addition, the liver is the principal metabolic organ of the body. This ability to develop the leading role in centralizing the metabolic needs of the body would be impaired in chronic liver diseases. Actually, in liver cirrhosis, the restructuration of the liver parenchyma by fibrosis blocks the portal flow and induces the creation of regeneration nodules, which are exclusively vascularized through the hepatic artery [45]. Therefore, the liver, an organ with double blood vascularization, is progressively transformed into an organ with simple vascularization that is principally arterial vascularization. The central metabolic deficit which leads to hepatic insufficiency implies the needed creation of peripheric compensatory mechanisms in which the mast cells could participate.

A critical function of the liver is the clearance of noxious substances of intestinal origin, like translocated gut microbiota that arrive via the portal vein in the liver. This function is principally made by the Kupffer cells that have an extremely effective phagocytic system since they represent approximately 80% of the phagocytic mononuclear system. Thus, Kupffer cells, the largest tissue resident macrophage population, are key for the maintenance of liver integrity, as well as the local initiation and resolution of innate and adaptive immunity [47]. Therefore, they are key modulators of immune responses at interface organs, such as the skin or gut. This phagocytic function, which is impaired in liver cirrhosis, could be taken over peripherally by the mast cells, and in particular by those located in the mesenteric lymph complex. Mast cells have direct phagocytic and indirect functions since their proteases effectively neutralize endogenous toxins and venoms [48,49]. Thus, proteases released by activated mast cells detox an array of toxic substances [49]. Moreover, mast cell proteases achieve an extracellular digestive function. Perhaps this mechanism effectively collaborates in the support of basic nutrient substrates either to their microenvironment cells or at long distance, then is transported by the lymphatic system.

In addition, mast cells orchestrate immune cell functions, including essential support for triggering T-cell expansion by dendritic cells and sometimes even acquisition of dendritic cell antigen-presenting molecules [48]. In this way, mast cells can prime tissues for adequate inflammatory responses and cooperate with dendritic cells in T-cell activation. Mast cells also produce antimicrobial peptides like cathelicidins in response to toll-like receptors mediated by pathogen sensing and interact with innate immune cells. Thus, the mast cells collaborate with T cells to mount host defenses against viral, bacterial, fungal, and parasitic infections, e.g. by secreting TNF-α to recruit neutrophils through Toll-like receptor 2-triggered degranulation, or provision of extracellular DNA traps [48,49]. Interstitial intestinal edema favors lymphatic drainage of noxious substances to the mesenteric lymph nodes. In this way mast cells recruited by these lymph nodes can act like scavengers of the toxic intestinal products [48,49]. Mast cells’ preformed mediators can trigger vascular responses, in particular vasodilatation and vessel permeabilization, resulting in tissue edema [49]. The mast cells located in the splanchnic lymphatic axis can even influence the liver fibrosis evolution through production and systemic release of exosomes [50]. The mast cell exosomes, thanks to their specific content of microRNAs and proteases, could also induce a systemic anti-inflammatory function as well as reduce the hepatic fibrogenesis. In this sense, the potential applications of exosomes as biomarkers and therapeutics for liver diseases has been suggested [51,52].

This lymphatic node detoxing function would be associated with a metabolic burst releasing reactive oxygen species (ROS) with the subsequent production of oxidative stress. However, the mast cells have a peroxisome content that performs antioxidant functions, thus reducing the deleterious effects of ROS. The peroxisomes are small organelles, which are basic for the development of a stress response [53]. ROS play a leading role among their key functions. In mammalian cells, the oxidative breakdown of amino acids and fatty acids catalyzed by oxidases results in the transfer of hydrogen. The substrates sent directly to oxygen thereby generate hydrogen peroxide (H_2_O_2_) [54] and then H_2_O_2_ that could suffer degradation by catalase into two H_2_O molecules. This important function is impaired in the liver diseases, which is why it is considered a key factor in the production of Non-alcoholic steatohepatitis (NASH) [55,56]. In summary, peroxisome derived activity in mast cells recruited in the lymphatic mesenteric complex would try to supply or minimize the deficient antioxidant functions of the diseased liver. Peroxisomal disorders are inherent to the hepatic dysfunction [57]. However, peroxisome proliferator-activated receptor-beta/delta (PPAR-β/δ) and PPAR-alpha with multiple anti-inflammatory effects could modulate the mast cell phenotype reducing the levels of inflammatory cytokines in liver chronic diseases [43,58,59].

The liver is involved in the production of multiple metabolic functions, including lipid metabolism. Thus, the liver plays a key role in the development of hepatic steatosis and metabolic syndrome [55,56]. This is the reason why the role of the mast cells in the lipid metabolism through their peroxisome ability for fatty acid beta-oxidation can reduce this liver deficit [59]. The peroxisomes are organelles that can control metabolic cell functions including lipid metabolism in association with mitochondria [58,60,61]. In mammalian cells, peroxisomes and mitochondria functionally cooperate in the oxidative degradation of fatty acids by beta-oxidation [62].

In the chronic hepatic insufficiency, the splanchnic inflammation produced by mast cells would induce hypoxia [63]. Consequently, in chronic liver diseases the metabolic activity is reduced. Therefore, the oxidative phosphorylation is also inhibited [64], and finally, the energetic support is limited. This fact would imply the progressive reduction of the specialized epithelial cell number of the hepato-intestinal axis that consumes great amounts of usable energy (Figure 2). This energy saving induced by mast cells could also favor the pentose phosphate pathway since, through the formation of ribose-5-phosphate, the synthesis of nucleotides increases [38]. In this way, the metabolism and survival of the gastrointestinal and hepatic epithelial stem cells would be prioritized. Consequently, it could explain how the disappearance of the lobulillar structure is replaced by regeneration nodules [45]. In the intestine, the metabolism of the crypt cells would be prioritized. The stem-cell niche, including the enteroendocrine cells, i.e., Paneth cells, which are cells-sensory sentinels, can be orchestrators of mucosal survival [65] when associated with mast cells. In addition, we’ve demonstrated the existence of goblet cell hyperplasia in portal hypertensive rats with mucus hyperproduction that have a protecting effect over the intestinal mucosa [66,67].

The coupling lymphatic-mast cell would represent a splanchnic defensive mechanism against the injury that produces portal hypertension and/or hepatic insufficiency. This coupling is formed structurally by an anatomical axis in which a poor oxygenated environment predominates (Figure 6). The intestine maintains a low baseline PO_2_ level due to high rates of metabolism, countercurrent blood flow, and the presence of a steep oxygen gradient across the luminal aspect of tissue surface. As a result, hypoxia exists even in the healthy, unperturbed intestinal mucosa [63]. This homeostatic situation of intestinal hypoxia aggravates in portal hypertension through the development of a splanchnic hyperdynamic circulation, and the creation of an anaerobic metabolism, among other factors [67,68]. Therefore, in portal hypertension and/or hepatic insufficiency, the return of the organism to this ancestral metabolic situation could aim to preserve the hepato-intestinal survival mechanisms (Figure 2).

## 4. Conclusions

In summary, the reduction of the hepatic metabolism in chronic liver diseases would induce its central metabolic, detoxing and antioxidant abilities, which are basically mediated by peroxisomes. As a result, peripherical structures could try to supply the abovementioned functions. In particular, the mast cell that behaves as a metabolic sensor in the hepatic insufficiency would increase its number and express its different phenotypes, which would coordinate to compensate the deficiencies caused by the hepatic insufficiency. Thus, the mast cells with their peroxisomes content would carry out these partially lost functions in the peripheric organs and tissues, especially in the splanchnic area. At last, it has been considered that the splanchnic blood vascular stasis not only occurs in liver diseases, but also in other situations. For instance, in the cases in which the intrabdominal pressure increases, like in obesity or a sedentary lifestyle, the splanchnic, venous stasis would produce a chronic low-grade gut-related inflammatory response by mechanotransduction. If so, a mast cell compensatory reaction takes place, which is associated with a splanchnic circulatory switch with increased development of a venous-lymphatic circulation at the expense of the arterial circulation.
